# A Paper-Based IL-6 Test Strip Coupled With a Spectrum-Based Optical Reader for Differentiating Influenza Severity in Children

**DOI:** 10.3389/fbioe.2021.752681

**Published:** 2021-10-06

**Authors:** Sheng-Wen Lin, Ching-Fen Shen, Ching-Chuan Liu, Chao-Min Cheng

**Affiliations:** ^1^ Institute of Biomedical Engineering, National Tsing Hua University, Hsinchu, Taiwan; ^2^ Department of Pediatrics, National Cheng Kung University Hospital, College of Medicine, National Cheng Kung University, Tainan, Taiwan; ^3^ Institute of Clinical Medicine, College of Medicine, National Cheng Kung University, Tainan, Taiwan; ^4^ Center of Infectious Disease and Signaling Research, National Cheng Kung University, Tainan, Taiwan

**Keywords:** influenza infection, interleukin-6, point-of-care testing, paper-based test strip, spectrum-based optical reader, children

## Abstract

Influenza virus infection is a major worldwide public health problem. Influenza virus infections are associated with a high hospitalization rate in children between the ages of 5 and 14. The predominant reason for poor influenza prognosis is the lack of any effective means for early diagnosis. Early diagnosis of severe illness is critical to improving patient outcome, and could be especially useful in areas with limited medical resources. Accurate, inexpensive, and easy-to-use diagnostic tools could improve early diagnosis and patient outcome, and reduce overall healthcare costs. We developed an interleukin-6 paper-based test strip that used colloidal gold-conjugated antibodies to detect human interleukin-6 protein. These complexes were captured on a paper-based test strip patterned with perpendicular T lines that were pre-coated with anti-human interleukin-6 antibodies. Applied serum samples interacted with these antibodies and presented as colored bands that could be read using a spectrum-based optical reader. The full-spectrum of the reflected light interleukin-6 protein signal could be obtained from the spectral optics module, and the standard could be used to quantitatively analyze interleukin 6 level in serum. We retrospectively evaluated 10 children (23 serum samples) with severe influenza virus infections, 26 children (26 serum samples) with mild influenza virus infections, and 10 healthy children (10 serum samples). Our system, the combined use of a paper-based test strip and a spectrum-based optical reader, provided both qualitative and quantitative information. When used with the optical reader, the detection limit was improved from a qualitative, naked-eye level of 400 pg/ml to a quantitative, optical reader level of 76.85 pg/ml. After monitoring serum interleukin-6 level via our system, we found a high correlation between our system results and those obtainable using a conventional sandwich enzyme-linked immunosorbent assay method (Rho = 0.706, *p* < 0.001). The sensitivity and specificity for differentiating between severe and mild influenza using our combined method (test strip coupled with optical reader) were 78.3 and 50.0%, respectively. When interleukin-6 was combined with serum C-reaction protein, the sensitivity and specificity were 85.7 and 95.5%, and the receiver operating characteristic area-under-the-curve was quite high (AUC = 0.911, *p* < 0.001). The potential advantages of our system, i.e., a paper-based test strip coupled with a spectrum-based optical reader, are as follows: 1) simple user operation; 2) rapid turnaround times–within 20 min; 3) high detection performance; and, 4) low-cost fabrication.

## Introductions

Influenza virus infection is a major worldwide public health problem. Seasonal infections with the common influenza virus strains (e.g., H1N1) are frequently resolved, but still cause high mortality. The annual morbidity rate caused by influenza is the highest in children, where morbidity is usually >30% (Diseases CoI, 2020; Hurwitz et al., 2000a). Children are also considered to be the main spreaders of influenza in the community (Hurwitz et al., 2000b; Reichert et al., 2001; Neuzil et al., 2002a). Studies have shown that the rate of hospitalization for influenza-related illnesses in infants and young children without underlying diseases is comparable to that of high-risk adults (Izurieta et al., 2000; Neuzil et al., 2002b; Chiu et al., 2002). In addition, it has been reported that influenza virus infection with a high hospitalization rate occurred in children between the ages of 5 and 14 years. A small percentage of these patients could develop more complicated and severe symptoms, e.g., elevated fever, violent dry cough, pneumonia, and acute respiratory distress syndrome, that require admission to an intensive care unit (Kamigaki and Oshitani, 2009; Hasegawa et al., 2010). The predominant reason for poor influenza prognosis is the lack of any effective means for early diagnosis. Early diagnosis of severe illness is critical to improving patient outcome, and could be especially useful in areas with limited medical resources. Accurate, inexpensive, and easy-to-use diagnostic tools could improve early diagnosis and patient outcome, and reduce overall healthcare costs.

Inflammation is a rapid and non-specific host defense mechanism that is strictly regulated by a network of inflammatory mediators. Inflammatory mediators include cytokines, such as interleukin-6 (IL-6) (Ryan and Majno, 1977; Feghali and Wright, 1997; Rouse and Sehrawat, 2010). IL-6 is a pleiotropic cytokine, which is significantly related to all aspects of the immune response, including inflammation. IL-6 is the main mediator of acute-phase reactions and fever (Gabay and Kushner, 1999). IL-6 is also associated with many pathogenic inflammatory states: increased levels of IL-6 are associated with sepsis severity and mortality (Dama et al., 1992), and IL-6 has been implicated in the cytokine storm following infection with avian influenza A H5N1 and severe responses to infection such as severe acute respiratory syndrome infection (Zhang et al., 2004; Huang et al., 2005; De Jong et al., 2006). Contrastingly, studies have also shown that IL-6 may play a role in regulating and limiting inflammation (Xing et al., 1998). Within the lung, IL-6 promotes lung inflammation, and IL-6 levels are related to the severity of acute respiratory distress syndrome as well as mortality (Headley et al., 1997; Lin et al., 2010). IL-6 levels have also been associated with symptom duration and severity in cases of seasonal influenza infection (Gentile et al., 1998; Fritz et al., 1999; Skoner et al., 1999). Thus, the role of IL-6 for diagnosing the progression of influenza warrants special attention.

Although conventional methods to gain information via the use of biomarkers are common, most examinations must be performed in hospitals and require time, expensive/complex equipment, and well-trained professionals to facilitate inspection and analysis. Based on current global medical trends, we predict that the future medical ecosystem will provide an increasing number of opportunities for improvement via the use of point-of-care (POC) tools at the bench and at bedside (Hudspeth and Morse, 2017; Nayak et al., 2017; Wang et al., 2020a; Hung et al., 2021). Rapid diagnostic tests are needed to help clinicians distinguish between critically ill and mildly ill influenza patients in a timely manner. To appropriately meet the requirements and demands of POC devices, these diagnostic devices must meet the following factors: 1) low sample consumption; 2) minimized user intervention; 3) rapid turnaround times; 4) extended storage and shelf life of reagents; 5) high sensor performance; and, 6) low fabrication cost. Further, the use of portable readout devices, cell-phone-based systems, and equipment-free systems are popular in developing countries and other resource-poor environments.

Here, we discuss the design, prototyping, and testing of a paper-based test strip that can detect IL-6 via lateral flow immunoassay (LFA), and can be qualitatively read with the naked eye (detection limit of 400 pg/ml). To increase the detection limit of the test strip, a small, light, and easy-to-use spectrum-based optical reader, a collaborative effort with SpectroChip Inc. in Taiwan, was integrated into the methodology in order to capture the reflection spectrum on the test strip for analysis. This research and our perspectives could provide options and impetus for the development of a POC device for influenza diagnosis with an eye toward easy, early diagnosis as a means of improving patient outcome. Our results may also be useful for the development and implementation of methods for determining influenza severity and optimizing treatment, which would be especially useful if applied to a pediatric population.

## Materials and Methods

### Patients and Samples

Patients with virologically confirmed influenza infection visiting National Cheng Kung University Hospital, Taiwan were recruited. Influenza infection was defined as having clinical presentation of acute respiratory disease, plus virological evidence of influenza infection, either through positive influenza viral antigen test (Flu A + B Rapid Antigen Test, BD Veritor™ Plus System, United States of America) or influenza virus isolated from respiratory specimens. The influenza infection cases were then further classified into mild or severe cases. Mild cases included those with only fever and respiratory symptoms, and severe cases presented lower respiratory tract infection, acute respiratory distress, hemodynamic instability, or severe extrapulmonary manifestation (myositis, encephalitis, seizure, or myocarditis). Children without fever, respiratory symptoms or any sign of acute infection within 1 week were enrolled as healthy controls. Serum samples from another infectious disease cohort, including patients with dengue virus, enterovirus, and *Mycoplasma pneumoniae* infections, were only used for correlation analysis between our IL-6 test strip protocol and ELISA methodology (n = 24; n = 83 with influenza infection cases). The protocol for this study was reviewed and approved by the Institutional Review Board (IRB) of National Cheng Kung University Hospital (IRB No: B-ER-102-345). Informed consent was obtained from each participating patient or the patient’s parents or guardian.

### Cytokine Assay

Soluble IL-6 concentration in serum samples was determined using monoclonal antibody enzyme-linked immunosorbent assay (ELISA) kits (D6050, R&D systems, United States). The minimum detectable concentration was 3.1 pg/ml. The intra- and interassay coefficients were less than 7%. The ELISA reader brand was Tecan (SunriseTM | Absorbance Microplate Reader). The experiment protocol and analyzing method all follow the standard protocol of the ELISA kit. We measured IL-6 concentration in all 83 serum samples.

### Lateral Flow Immunoassay

This paper test strip (created in cooperation with Taiwan Hygeia Touch Inc.) used colloidal gold-conjugated anti-IL-6 antibody (ARG21446, arigo Biolaboratories Corp. Hsinchu, Taiwan) to detect human IL-6 antigen. Based on the colorimetric analysis of each test combination, an optimization test was performed to determine the most suitable IL-6 concentration and gold nanoparticle size for conjugation. The test strip was pre-coated with capture reagent and sprayed to generate vertical lines containing anti-IL-6 antibody (ARG21444, arigo Biolaboratories Corp. Hsinchu, Taiwan) bound to 15 nm-diameter gold colloid. The C line of the nitrocellulose strips was also coated with mouse anti-human IgG antibody (ARG21957, arigo Biolaboratories Corp. Hsinchu, Taiwan) and an equal amount of quality control antibody. When the applied sample flowed along the strip, the human IL-6 antigen present in the sample formed an antigen complexed with the colloidal gold-labeled anti-IL-6 antibody. These complexes are then captured in the vertical T-line of the test strip, where they appeared as colored strips.

### Reflectance Spectral Analysis

The spectrum analyzer (in cooperation with Taiwan SpectroChip Inc.; Taiwan FDA: MD (I)-008090 and US FDA: 3017810861) was equipped with a cassette tape designed to accept the paper-based test strip and could detect the IL-6 antigen reflectance spectrum from the test paper. This device provided continuous spectrum results and captured high-resolution reflectance spectrum values from the test strip test line (T line). The spectrum reader provided high resolution (3–5 nm) results over a wide spectral range (300–1,100 nm). The principle of machine detection is to scan with white light. When the red line was scanned, the colloidal gold absorbed the spectrum between 430 and 600 nm and reflected the remaining light that was not absorbed by the colloidal gold. Colloidal gold does not absorb 650 nm light, so the red lines of different shades produced little change at 650 nm, which is approximately equal to 1.

The main reflection wavelengths detected by this spectrum reader were 430 and 600 nm (for samples with lower IL-6 concentration), and the main reference wavelength was 650 nm. The ratio of the minimum reflectance to the spectrum at the reference wavelength was used to calculate the α value: α = Reflectance (650 nm)/Reflectance (the minimum value in the range of 430–600 nm).

In this formula, α refers to the color reflection value of the optical scanning IL-6 antigen rapid screening test strip. The higher the α value, the stronger the reflection color intensity of the IL-6 complex coupled with the colloidal gold antibody, which indicated higher the IL-6 concentration.

### Limit of Detection and Limit of Quantification

The LOD and LOQ were estimated based on the average of the blank α value, the standard deviation of the blank α value, the slope of the calibration curve (analytical sensitivity) and the defined confidence factor, using the following calculations:

LOD = Blank (mean) + 3 x Blank (standard deviation)

LOQ = Blank (mean) + 10 x Blank (standard deviation)

**FIGURE 1:**
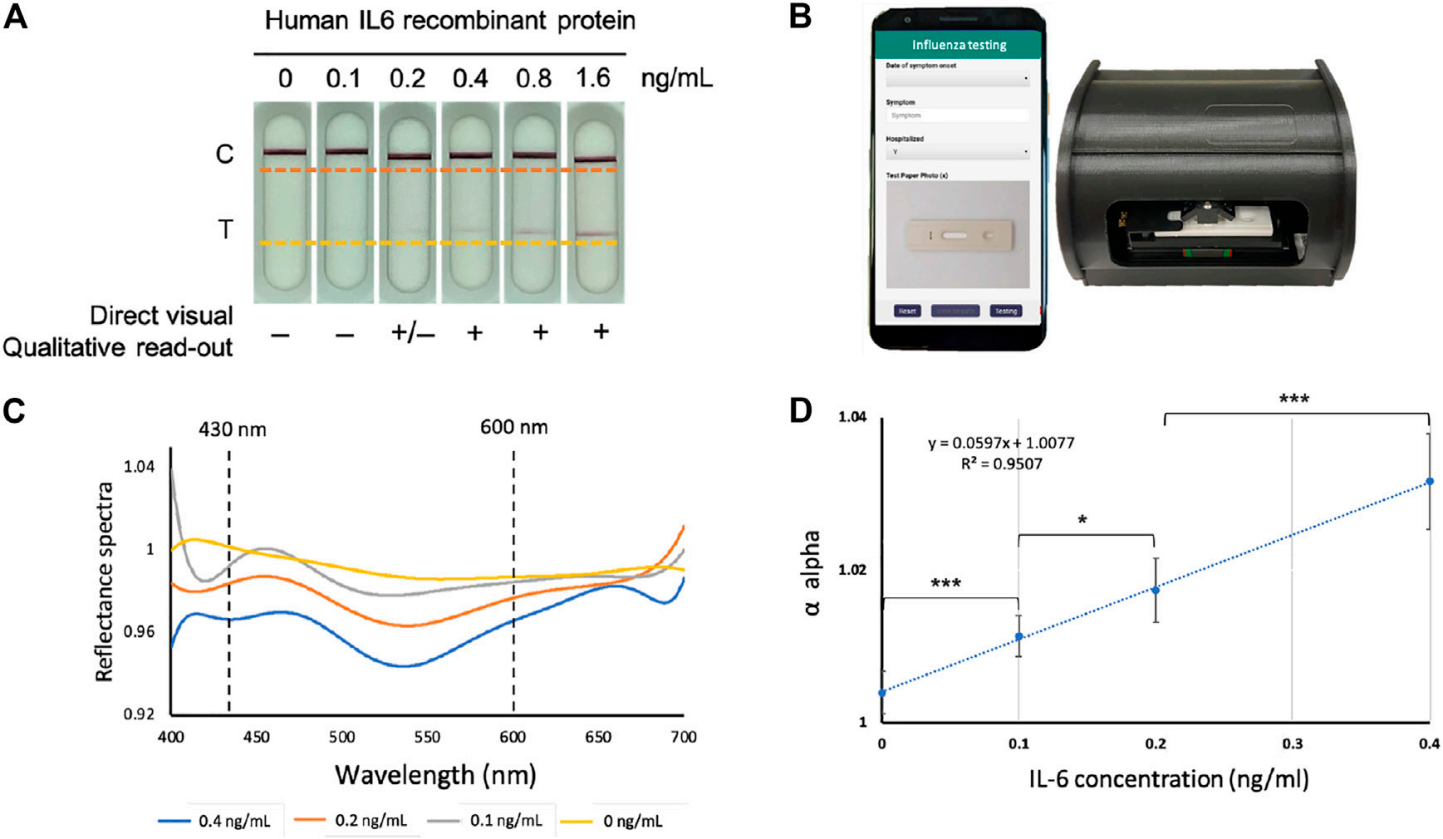
The IL-6 paper-based test strip and spectrum-based optical reader. **(A)** The IL-6 test strips loaded with pre-determined amounts of purified IL-6 protein. **(B)** Spectrum-based optical reader and mobile-phone which could connect to the reader (in collaboration with SpectroChip Inc., Taiwan; Taiwan FDA: MD **(I)**-008090 and U.S. FDA: 3017810861). **(C)** The reflectance spectra of the predetermined amounts of purified IL-6 protein in standard scale. **(D)** Linear regression for the α value of IL-6 protein at concentrations of 0, 0.1, 0.2, 0.4 pg/ml to determine the limit of detection (LOD) and limit of quantification (LOQ), *Y*-axis; α value, *X*-axis; IL-6 concentration based on test strip coupled with optical reader, *p < 0.05, ***p < 0.001.

In this calculation, the average blank α value was 1.00395, the standard deviation of the blank α value was 0.00278, the blank coefficient of variation was 0.27%, and the 95% confidence interval was 99.6–100%. LOD was estimated by using the average value of blank α value plus 3 times the standard deviation of the average value of blank α value, using the following equation in Figure 1: y = 0.0597x + 1.0077 (y, α value; x, IL-6 concentration). Therefore, the α value of LOD was equal to 1.0122,881, and the concentration of LOD was 76.85 pg/ml. The same calculation was applied to determine LOQ.

### Statistical Analysis

The Wilcoxon rank sum test and the Wilcoxon signed rank test were used to check data and distinguish severe cases, mild cases, and healthy controls. A *p*-value less than 0.05 was considered statistically significant. The receiver operating characteristic (ROC) area under the curve (AUC) were used to evaluate the diagnostic ability of IL-6. In order to evaluate the correlation between this paper-based test strip/optical reader combination and ELISA, we used Spearman’s rank correlation coefficient and the Bland-Altman chart.

## Results

### Clinical Characteristics of Study Cohort

A total of 36 influenza infected patients (10 severe cases/23 serum samples, and 26 mild cases/26 serum samples) and 10 healthy individuals (10 serum samples) were recruited. The clinical characteristics and investigative findings are provided in Supplementary Table S1. Among the influenza patients, two of them had serial serum samples collected during the course of their illness. Most patients with mild disease characteristics only visited the outpatient clinic or the emergency department, but two of them were hospitalized. Among the severe influenza patients, most required intensive care, except one pneumonia case, which was cared for in the general ward. Age and gender were not significantly different across disease severity levels.

### The Spectrum-Based Optical Reader Provided High-Performance Spectral Analysis for the Influenza IL-6 Test Strip

As shown in Figure 1A, there were bands on the control (C) line on all test strips, verifying the test for various IgG concentrations. The band intensity of the IL-6 (T) line decreased as the concentration of purified recombinant IL-6 protein decreased, and the band became almost invisible on test strips loaded with IL-6 at a concentration lower than 0.4 ng/ml. Figure 1B provides a picture of a spectrum-based optical reader that can be activated through a mobile APP. In this quantitative platform system, a spectrometer was used to analyze LFA test color intensity, where the presence of IL-6 was based on the cutoff value of the spectral intensity. The LOD needed to be determined to evaluate the performance of the platform system. To this end, the IL-6 test strip was loaded with various predetermined purified amounts of IL-6 in the serum (0, 0.1, 0.2, and 0.4 ng/ml) to obtain continuous reflectance spectra (from wavelength 430–600 nm), and the data from six replicates were combined for analysis.

The percentage of the reflectance spectrum is negatively correlated with the number of antibody-antigen complexes, so it decreases as IL-6 concentration increased. Here, we found that the reflectance spectrum of IL-6 was well separated at about 540 nm (Figure 1C). The reflectance spectrum was used to obtain the α value, which was used to construct the IL-6 concentration standard curve of the reflectance. As shown in Figure 1D, after fitting the α values for each concentration, the linear regression provided an *R* ^2^ value of 0.9507. Based on this regression model, the LOD and limit of quantification (LOQ) were 76.85 pg/ml and 402.71 pg/ml, respectively. Based on these results, the platform provides an excellent analytical sensitivity (i.e., the limit of detection) for the detection of trace levels of human IL-6 protein.

### Correlation Between the IL-6 Test Strip System and Conventional ELISA Methodology

**FIGURE 2:**
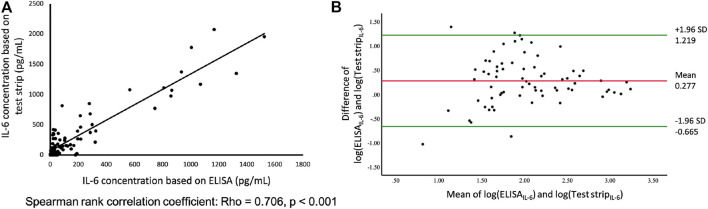
**(A)** Comparison of paper-based test strip and ELISA for IL-6 assays using serum from patients, including influenza, enterovirus, Mycoplasma, dengue, n = 83. *Y*-axis; IL-6 concentration measured by test strip. *X*-axis; IL-6 concentration measured by ELISA. **(B)** Bland and Altman plot of log-transformed data. The differences between the IL-6 based on test strip and ELISA (log transformation) in relation to the mean of the two measurements (log transformation), n = 83. Green lines indicate the limits of agreement (±1.96 SD).

We selected 83 patients, all of which were children that had different illness severities. We then measured each patient’s serum IL-6 concentration using two tests: 1) the IL-6 test strip coupled with a spectrum-based optical reader; and, 2) enzyme-linked immunosorbent assay (ELISA). As shown in Figure 2A, the correlation between the IL-6 concentration results based on our test strip system and the concentration based on ELISA methodology were highly relevant and statistically significant (Rho = 0.706, *p* < 0.001).

To validate the agreement between these two assays, we used the Bland-Altman analysis to assess any deviations between the mean differences and estimate the interval of agreement (95%) between the test strip system and ELISA. The Bland-Altman diagram simply represents the comparison of each difference between the two pairing methods with the measured average, as shown in Figure 2B. After logarithmically transforming the original data of the concentration of IL-6 based on test strip and ELISA results, the Shapiro-Wilk test was used to determine normality (*p* = 0.46) (Euser et al., 2008; Giavarina, 2015). The mean difference of the two assays was 0.277, and the limits of agreement ( ± 1.96 standard deviation) were 1.219 and -0.665. The width of the limits of agreement was 1.884, which included most of the data points. There was no trend between the difference and the mean, but the difference between the two methods was more variable during the mean from 1.5 to 2.5.

### Capacity of the IL-6 Test Strip to Distinguish Influenza Severity

**FIGURE 3:**
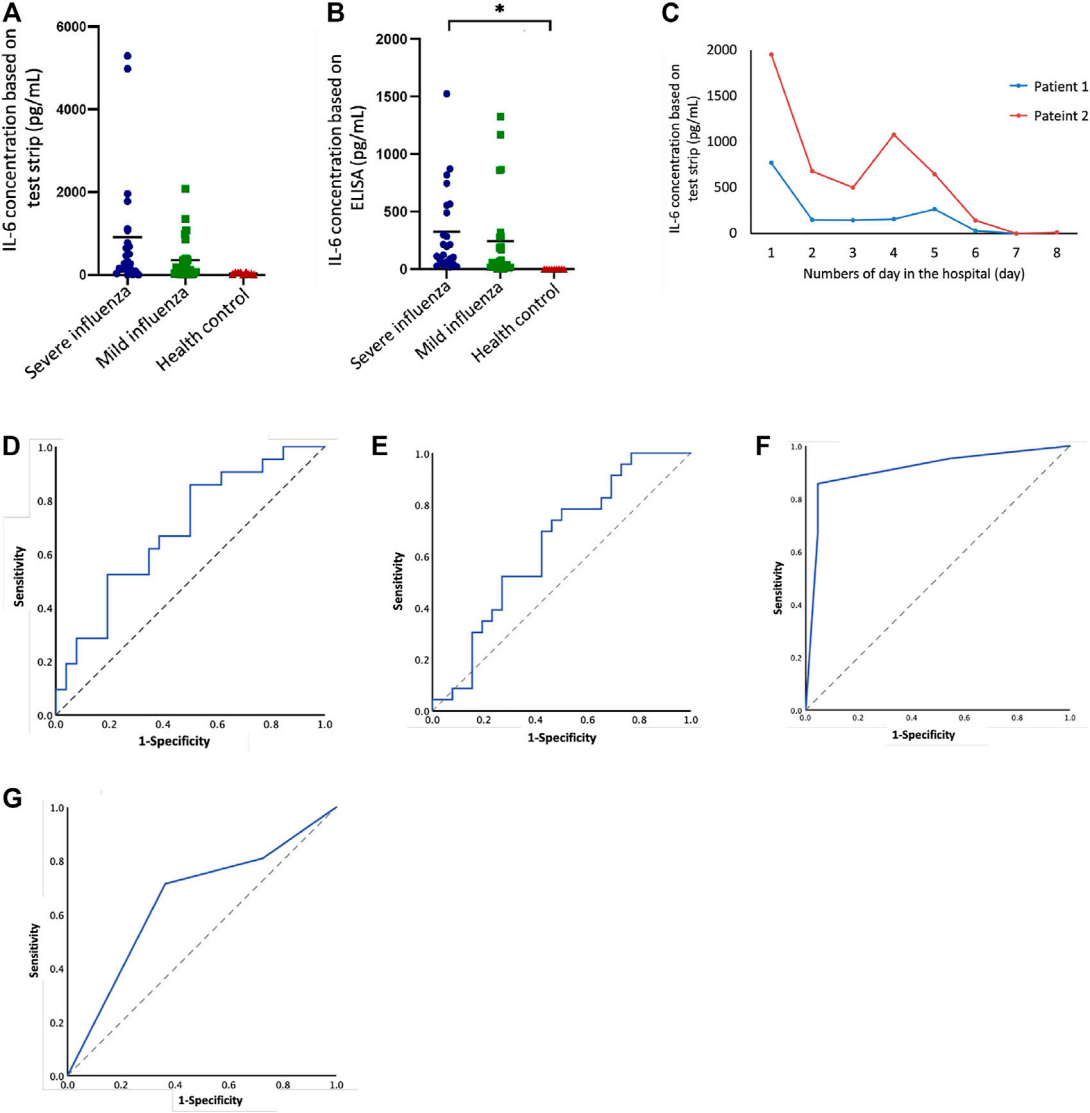
IL-6 levels in severe cases and mild cases of influenza in children based on test strip and ELISA. **(A)** IL-6 (test strip) concentration between different groups: severe influenza (n = 23), mild influenza (n = 26), health control (n = 10). **(B)** IL-6 (ELISA) concentration between different groups: severe influenza (n = 23), mild influenza (n = 26), health control (n = 10). There was a statistically significant difference between severe cases and healthy controls (*p* < 0.05). **(C)** Follow-up IL-6 test strip concentrations compared to number of days in the hospital for two patients. **(D)** ROC curve of IL-6 concentration (test strip), AUC = 0.69, *p* = 0.026. **(E)** ROC curve of IL-6 concentration (ELISA), AUC = 0.64,*p* = 0.092. **(F)** ROC curve of IL-6 concentration (test strip) combined with CRP, AUC = 0.911, *p* = 0.00. (G) ROC curve of IL-6 concentration (ELISA) combined with CRP, AUC = 0.654, *p* = 0.085.

Based on the IL-6 level as determined by test strip assay (Figure 3A), severe influenza cases tended to have higher IL-6 level compared to mild cases, but the difference did not reach statistical significance (*p* = 0.071). The IL-6 level results provided by the conventional sandwich ELISA method (Figure 3B) displayed a similar trend, however, IL-6 level among severe cases was significant higher compared to that of healthy controls (*p* < 0.05). Although there was no significant difference in IL-6 level between severe and mild influenza cases, two cases with serial serum samples demonstrated that IL-6 level correlated with disease progression (Figure 3C and Supplementary Figure S1). The first patient was a 4 year-old boy who developed bilateral bacterial pneumonia, respiratory failure, and shock during hospitalization. He was intubated for ventilation support and received inotropic agents for hemodynamic instability on the first day when his measured IL-6 level exceeded 700 pg/ml. After treatment, the patient discontinued inotropic treatment on day 4, and was extubated on day 6. During this period, IL-6 level declined to 150–200 pg/ml. When the patient was stabilized and further transferred to the general ward, IL-6 level dropped to 0–50 pg/ml. The second patient, an 8 year-old boy, developed necrotizing pneumonia with pleural effusion and respiratory failure. This patient demonstrated rapidly deteriorated respiratory condition and was intubated on the first day, when his IL-6 level reached 1,500 pg/ml. Following chest tube drainage of pleural effusion and antimicrobial treatment, this patient’s IL-6 level dropped on days 2 and 3. This patient’s IL-6 level rose temporally after video-assisted thoracotomy debridement for his empyema, but then further declined gradually after his condition stabilized. Two days before transferal to the general ward, his IL-6 level dropped to below 20 pg/ml.

### The Receiver Operating Characteristic Curve

The ROC curve could be useful for summarizing the performance of each classifier into a single measure. It shows the trade-off between sensitivity (or TPR) and specificity (1–FPR). As shown in Figures 3D,E, the area under the curve (AUC) values for both the test strip and ELISA assays were 0.69 and 0.64 (*p* < 0.05 and *p* > 0.05), respectively. The cut-off value according to the test strip ROC curve was 140.4 pg/ml, and the sensitivity and specificity were 78.26 and 50.0% (Table 1). We calculated several cutoff values suggested by studies (Oh et al., 2002; Sato et al., 2009; Chiaretti et al., 2013), such as 100 and 150 pg/ml, and found that the sensitivity and specificity were 78.26, 65.22, 55.56, and 63.89%, respectively. According to the ELISA ROC curve, the cut-off value was 60.78 pg/ml, and the sensitivity and specificity were 78.26 and 63.89%. The cutoff values, 100 and 150 pg/ml, suggested by ELISA studies do not seem to be very good, and respective sensitivity and specificity were 65.22, 52.17, 69.44, and 69.44%, respectively. In addition to examining IL-6 alone, we looked at the combination of IL-6 and serum C-reactive protein (CRP) to assess influenza severity. As shown in Figures 3F,G & Supplementary Figure S2, the AUC measured using the test strip and ELISA were 0.911 and 0.664 (*p* < 0.05 and *p* > 0.05). Combining IL-6 with CRP provided a high AUC in the ROC curve for the test strip results. The sensitivity and specificity for IL-6, when combined with CRP, were 85.71 and 95.45%; the sensitivity and specificity for IL-6, when combined with CRP, were 71.43 and 63.64%. Of the other available clinical information, i.e., the number of days in the hospital, the number of days in the ICU, and highest body temperature, the first two values (number of days in the hospital and the number of days in the ICU) correlated with test-strip IL-6 concentration (statistically significant; *p* < 0.05 and *p* < 0.01) (Table 2).

**TABLE 1:** Charecteristics of tests based on different cutt-off values.

	Cutt-off value	Sensitivity (%)	Specificity (%)	PPV	NPV
IL-6 (Test strip)	100 pg/ml	78.26	55.56	52.94	80.00
	140.4 pg/ml	78.26	50.00	58.06	72.22
	150 pg/ml	65.22	63.89	53.57	74.19
IL-6 (ELISA)	60.78 pg/ml	78.26	63.89	58.06	82.14
	100 pg/ml	65.22	69.44	57.69	75.76
	140.4 pg/ml	52.17	69.44	52.17	69.44
	150 pg/ml	52.17	69.44	52.17	69.44
CRP	30.85 pg/ml	80.77	95.24	95.45	80.00
IL-6 (Test strip) + CRP	140.4 pg/ml + 30.85 pg/ml	85.71	95.45	94.74	87.50
IL-6 (ELISA) + CRP	60.78 pg/ml + 30.85 pg/ml	71.43	63.64	65.22	70.00

**TABLE 2:** Correlation between IL-6 and other clinical information based on both test strip and ELISA results.

Correlation	CRP	Total number of days in hospital	Total number of days in ICU	Highest temperature
IL-6 (Test strip)	Rho = 0.179 (*n* = 43)	Rho = 0.433** (*n* = 36)	Rho = 0.396* (*n* = 35)	Rho = 0.275 (*n* = 35)
IL-6 (ELISA)	Rho = 0.211 (*n* = 43)	Rho = 0.302 (*n* = 36)	Rho = 0.301 (*n* = 35)	Rho = 0.24 (*n* = 35)

*The correlation is significant at the 0.05 level (two-tailed). **The correlation is significant at the 0.01 level (two-tailed).

### The Workflow of a Paper-Based Test Strip Coupled With a Spectrum-Based Optical Reader

**FIGURE 4:**
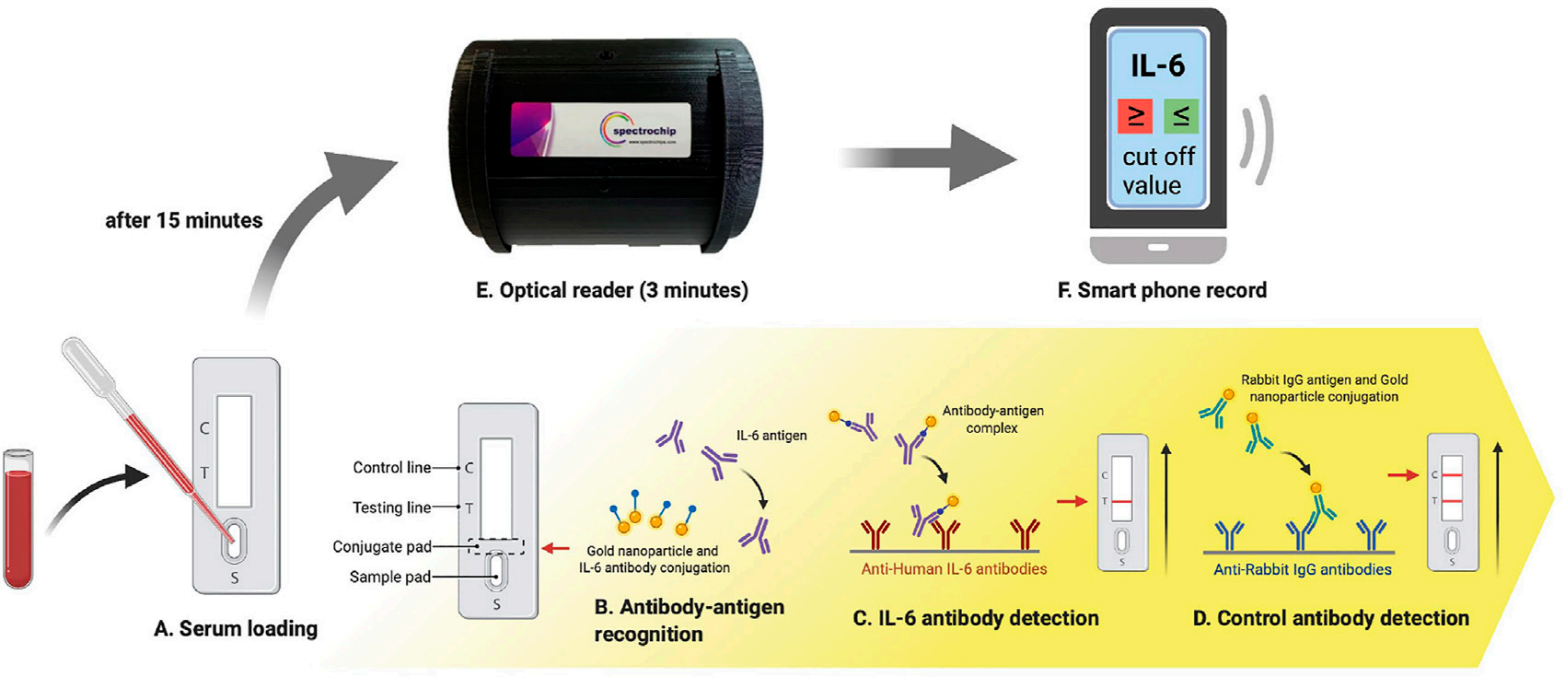
The IL-6 test strip workflow coupled with a spectrum-based optical reader. This new spectrum analyzer platform system requires only 0.1 ml of blood serum to be added to the test strip, and provides results in 15 min. The test strip is placed in a spectrometer for quantitative spectral analysis. This scan takes approximately 3 min to complete. Automatic scanning of the rapid test strip is activated with an APP. Full-spectrum antibody reflex optical signals are acquired from the spectral optical module to analyze IL-6 full-spectrum antibody distribution and concentration with standard quantification.

In order to provide a rapid, simple, precise, and inexpensive device, we designed an easy-to-use POC system. As shown in Figures 4A–D, the principle of the paper-based test strip to detect the IL-6 was a lateral flow immunoassay that provided visible, qualitative results after 20 min. Scanning the test strip with a light and small optical reader facilitated the acquisition of quantitative results. This reader was activated using a mobile phone APP (Figures 4E,F).

## Discussion

More than half (55%) of all children hospitalized for influenza have underlying respiratory diseases, including asthma (27%) (Xu et al., 2019). When a patient arrives in the emergency room with influenza-like symptoms and these underlying diseases appear, in addition to detecting the type of influenza, it is even more critical to perform diagnostic POC tests as soon as possible to predict the potential process of the influenza to promote rapid and accurate clinical decision-making. In addition, the symptoms and severity of flu vary widely. Some patients show no signs of illness, while others require hospitalization. The IL-6 test strip system may be used to inform diagnostic and treatment decisions for patients in clinical settings, including whether to prescribe antiviral medications. In periods of peak demand, the timely implementation of infection control and treatment of influenza, can significantly reduce the impact on hospital resources and patient management. Therefore, there is a great need for serological testing methods that can be widely distributed and detect current/past infections. Among a series of potential serological testing methods, lateral flow immunoassay is ideal for large-scale screening and POC testing. However, the readings from such devices are mostly qualitative (yes or no) and are generally considered not sensitive enough to diagnose serious infections such as influenza. In order to provide sensitive quantitative capabilities for lateral flow immunoassays, we integrated a newly designed spectrum-based optical reader into our IL-6 test strip program. Using this analysis platform, the detection limit of human IL-6 protein can reach 76.85 ng/ml. Although the limit of detection of the IL-6 test strip system is not as sensitive as the ELISA used in this study (3.1 pg/ml), it is sufficient to distinguish between the mild and severe influenza cases in children.

In this study, compared with mildly affected patients, severely affected patients had higher IL-6 concentrations, but the difference was not statistically significant. There may be some other factors we need to discuss, for example, the number of days that a patient had a fever before admission might affect the process of the disease, the immune system of children is not mature, and there are different type of influenza, etc. (Oh et al., 2002; Sato et al., 2009; Simon et al., 2015) In the future, to make the entire study more complete, we still have some areas for improvement, such as large-scale collection of patient samples to verify sensitivity and specificity, comparing various ELISA brands with our platform to determine whether different brands of ELISA will affect the result. We could also combine the commercially available CRP test strip (Brouwer and van Pelt, 2015; Lin et al., 2016; Eley et al., 2018; Althaus et al., 2019; How, 2020; Tsounidi et al., 2021) with our IL-6 test strip system. This combination would be a more practical approach, which not only improves the diagnostic sensitivity and specificity while using two biomarkers instead of using single biomarkers, but also reduces the entire test time and cost.

IL-6 may be considered a potentially useful biomarker for influenza testing, and its significance as a biomarker may be further underscored by its associated rise in patients with encephalopathy (Aiba et al., 2001; Oh et al., 2002; Chiaretti et al., 2013). To date, several chemiluminescence immunoassay analyzers can be used for influenza diagnosis. Although these products may be better at detecting the presence or absence of influenza, they are not suitable for predicting the course or severity of the disease. In addition, gold nanoparticles have a high affinity for sulfhydryl (-SH) groups, so they can easily combine to various proteins (including antibodies). The use of gold nanoparticles can also improve the stability of the antigen and extend the shelf life. Moreover, our spectrum-based optical reader is small and light, it is more available to be installed in each clinic or ward to monitor and track of the disease process. The most important thing is that it is also less expensive. Furthermore, several optical readers based on smartphones have been previously reported for the quantification of lateral flow immunoassays (Hsu et al., 2014; Yang et al., 2016; Jalal et al., 2017; Mathaweesansurn et al., 2017; Xiao et al., 2018). Most test strip readers use image analysis, which can only scan the intensity for quantification; our reader is a spectrometer that can scan out colloidal nanogold reflectance spectrum and use the characteristic wave of the spectrum for quantification. Both approaches provide quantified results, but the spectrometer can also provide qualitative results and can better distinguish false positives caused by non-colloidal gold. Hence, our platform is not only easy to operate, but also highly sensitive for detecting of human IL-6 protein. We believe that our system could be used to help clinicians make influenza treatment decisions, and could potentially be used for guiding treatment for COVID-19 (Wang et al., 2020b; Gao et al., 2020; Han et al., 2020; Liu et al., 2020; Yang et al., 2020).

## Summary

To conclude, the advantages of our system, i.e., a paper-based analytical device coupled with a spectrum-based optical reader, are as follows: 1) simple user operation; 2) rapid turnaround times–within 20 min; 3) high detection performance; 4) low-cost fabrication; and, 5) small size, which saves workbench space and facilitates use in any medical environment. In addition, our system may contribute the following to the medical arena: 1) promotion of effective patient management; 2) assistance in targeted antiviral therapy and antibiotic management; 3) facilitation of immediate infection control measures; and, 4) reliable early-stage disease detection that could reduce overall medical costs (Bonner et al., 2003).

## Data Availability

The datasets presented in this study can be found in online repositories. The names of the repository/repositories and accession number(s) can be found in the article/Supplementary Material.
