# Corrigendum: A Paper-Based IL-6 Test Strip Coupled With a Spectrum-Based Optical Reader for Differentiating Influenza Severity in Children

**DOI:** 10.3389/fbioe.2021.802615

**Published:** 2021-12-15

**Authors:** Sheng-Wen Lin, Ching-Fen Shen, Ching-Chuan Liu, Chao-Min Cheng

**Affiliations:** ^1^ Institute of Biomedical Engineering, National Tsing Hua University, Hsinchu, Taiwan; ^2^ Department of Pediatrics, National Cheng Kung University Hospital, College of Medicine, National Cheng Kung University, Tainan, Taiwan; ^3^ Institute of Clinical Medicine, College of Medicine, National Cheng Kung University, Tainan, Taiwan; ^4^ Center of Infectious Disease and Signaling Research, National Cheng Kung University, Tainan, Taiwan

**Keywords:** influenza infection, interleukin-6, spectrum-based optical reader, point-of-care testing, paper-based test strip, children

In the original article there was an error in the **Funding** statement. The correct number for “Ministry of Science and Technology, Taiwan” is “MOST 110-2628-E-007-003.” The correct **Funding** statement appears below. Additionally, there were mistakes present in the captions for Figures 1–4, these have been corrected and the correct captions appear below.

**FIGURE 1:**
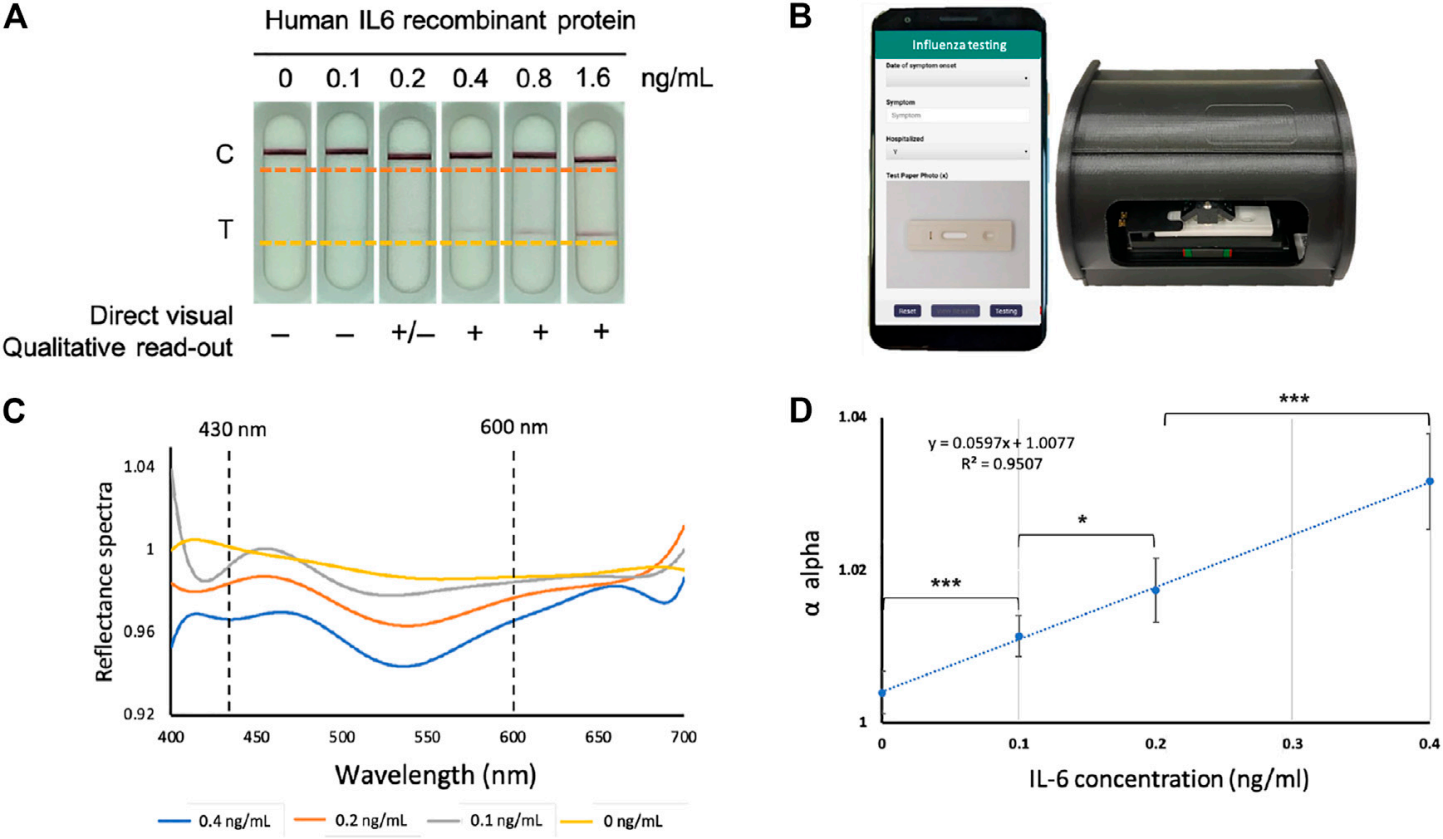
The IL-6 paper-based test strip and spectrum-based optical reader. **(A)** The IL-6 test strips loaded with pre-determined amounts of purified IL-6 protein. **(B)** Spectrum-based optical reader and mobile-phone which could connect to the reader (in collaboration with SpectroChip Inc., Taiwan; Taiwan FDA: MD (I)-008090 and U.S. FDA: 3017810861). **(C)** The reflectance spectra of the predetermined amounts of purified IL-6 protein in standard scale. **(D)** Linear regression for the α value of IL-6 protein at concentrations of 0, 0.1, 0.2, 0.4 pg/ml to determine the limit of detection (LOD) and limit of quantification (LOQ), *Y*-axis; α value, *X*-axis; IL-6 concentration based on test strip coupled with optical reader, *p < 0.05, ***p < 0.001.

**FIGURE 2:**
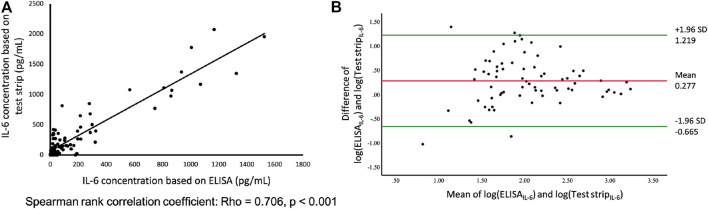
**(A)** Comparison of paper-based test strip and ELISA for IL-6 assays using serum from patients, including influenza, enterovirus, *Mycoplasma*, dengue, n = 83. *Y*-axis; IL-6 concentration measured by test strip. *X*-axis; IL-6 concentration measured by ELISA. **(B)** Bland and Altman plot of log-transformed data. The differences between the IL-6 based on test strip and ELISA (log transformation) in relation to the mean of the two measurements (log transformation), n = 83. Green lines indicate the limits of agreement (±1.96 SD).

**FIGURE 3:**
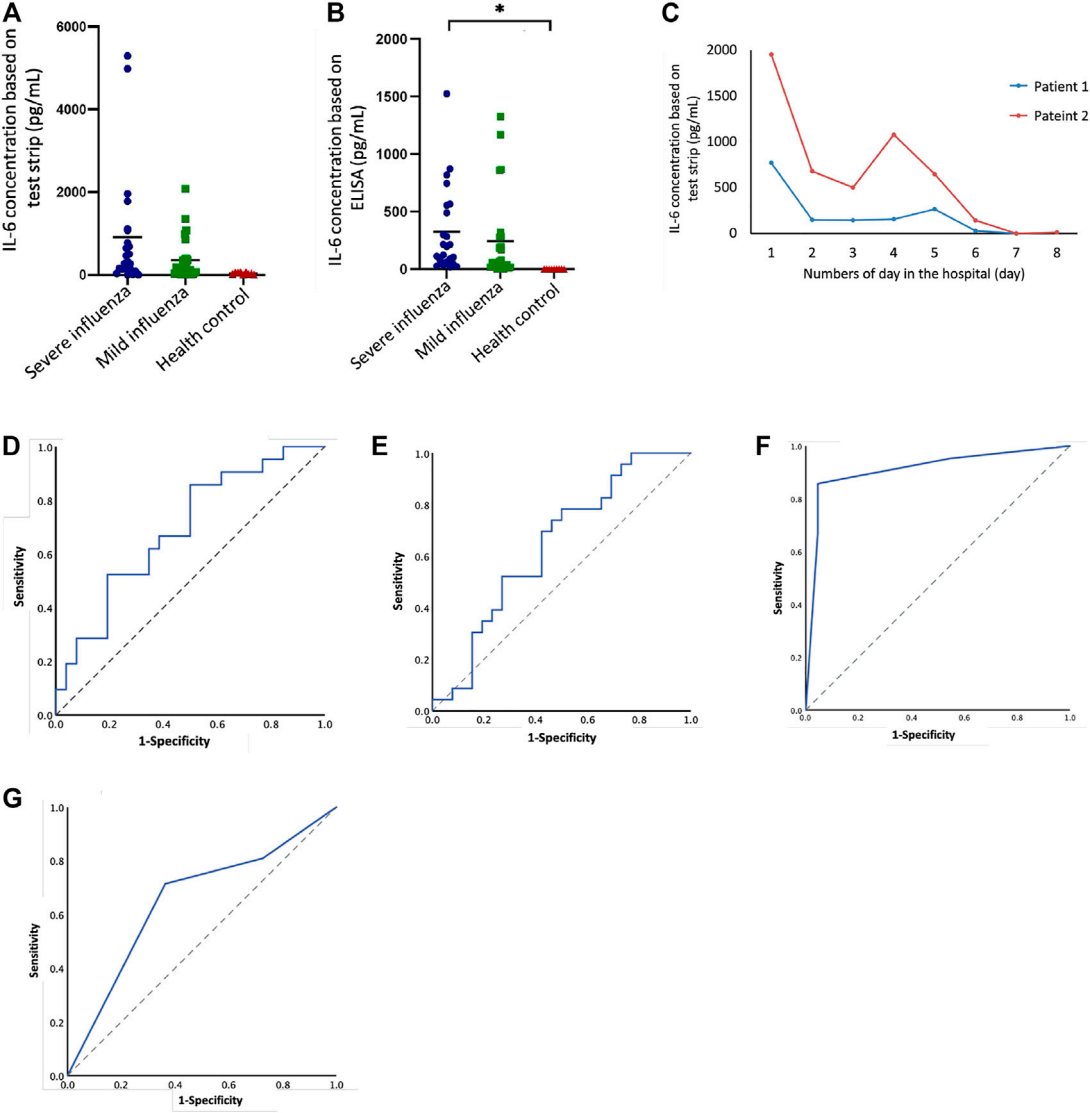
IL-6 levels in severe cases and mild cases of influenza in children based on test strip and ELISA. **(A)** IL-6 (test strip) concentration between different groups: severe influenza (n = 23), mild influenza (n = 26), health control (n = 10). **(B)** IL-6 (ELISA) concentration between different groups: severe influenza (n = 23), mild influenza (n = 26), health control (n = 10). There was a statistically significant difference between severe cases and healthy controls (*p* < 0.05). **(C)** Follow-up IL-6 test strip concentrations compared to number of days in the hospital for two patients. **(D)** ROC curve of IL-6 concentration (test strip), AUC = 0.69, *p* = 0.026. **(E)** ROC curve of IL-6 concentration (ELISA), AUC = 0.64, *p* = 0.092. **(F)** ROC curve of IL-6 concentration (test strip) combined with CRP, AUC = 0.911, *p* = 0.00. **(G)** ROC curve of IL-6 concentration (ELISA) combined with CRP, AUC = 0.654, *p* = 0.085.

**FIGURE 4:**
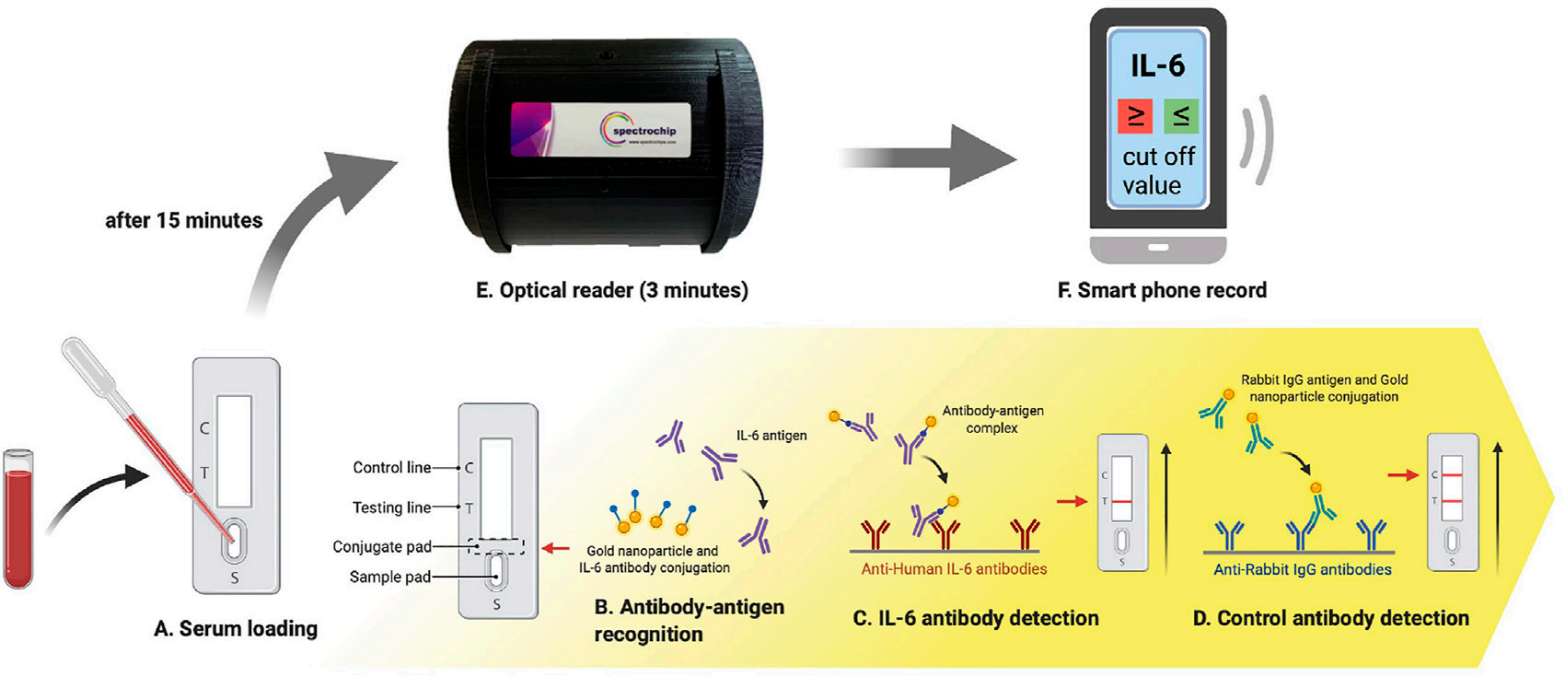
The IL-6 test strip workflow coupled with a spectrum-based optical reader. This new spectrum analyzer platform system requires only 0.1 ml of blood serum to be added to the test strip, and provides results in 15 min. The test strip is placed in a spectrometer for quantitative spectral analysis. This scan takes approximately 3 min to complete. Automatic scanning of the rapid test strip is activated with an APP. Full-spectrum antibody reflex optical signals are acquired from the spectral optical module to analyze IL-6 full-spectrum antibody distribution and concentration with standard quantification.

The authors apologize for these errors and state that they do not change the scientific conclusions of the article in any way. The original article has been updated.

