# Supplementary material for: A Paper-Based IL-6 Test Strip Coupled With a Spectrum-Based Optical Reader for Differentiating Influenza Severity in Children
Source: Front Bioeng Biotechnol. 2021 Oct 6;9:752681. doi: 10.3389/fbioe.2021.752681 (PMC8527092; doi:10.3389/fbioe.2021.752681)
Supplement: Supplementary file 1 [file Table1.DOCX]

**Supplementary Materials**

**Table 1. Patient characteristics.**

| Number | Sex | Age | Influenza | Total number of days in hospital | Total number of days in ICU | Highest temperature (^o^C) | CRP (mg/L) | IL-6 (ELISA, pg/mL) | IL-6 (Test strip, pg/mL) |
| --- | --- | --- | --- | --- | --- | --- | --- | --- | --- |
| 1 | F | 4.8 | Mild | 4 | 0 | 38.5 | <7 | 65 | 137.697 |
| 2 | M | 0.8 | Mild | 3 | 0 | 39 | 16.9 | 36.85 | 271.50 |
| 3 | F | 10.9 | Mild | 0 | 0 | 39.5 | 8.2 | 284.69 | 372.39 |
| 4 | M | 4.2 | Mild | 0 | 0 | 39.6 | 7 | 319.80 | 212.35 |
| 5 | F | 6.2 | Mild | 0 | 0 | 39.9 | 7.4 | 15.84 | 121.07 |
| 6 | F | 4.4 | Mild | 0 | 0 | 40 | 19.8 | 278.31 | 850.78 |
| 7 | F | 2.0 | Mild | 0 | 0 | 38 | - | 1325.10 | 1349.97 |
| 8 | M | 1.5 | Mild | 0 | 0 | 39 | 54 | 168.22 | 156.93 |
| 9 | F | 2.5 | Mild | 0 | 0 | 39 | 8.4 | 52.80 | 51.85 |
| 10 | M | 12.5 | Mild | 0 | 0 | 38 | 7.5 | 0.00 | 18.29 |
| 11 | M | 2.2 | Mild | 0 | 0 | 40.6 | <7 | 865.08 | 1069.42 |
| 12 | M | 3.7 | Mild | 0 | 0 | 38 | 12 | 10.25 | 0.00 |
| 13 | F | 1.7 | Mild | 0 | 0 | 39 | 13.9 | 859.59 | 975.88 |
| 14 | M | 13.5 | Mild | 0 | 0 | 39.2 | 26.4 | 1167.00 | 2076.72 |
| 15 | M | 3.6 | Mild | 0 | 0 | 38.2 | <7 | 194.01 | 394.70 |
| 16 | M | 4.8 | Mild | 0 | 0 | 38 | - | 2.81 | 68.82 |
| 17 | F | 8.9 | Mild | 0 | 0 | 36.7 | - | 18.23 | 37.10 |
| 18 | M | 16.1 | Mild | 0 | 0 | 38.6 | - | 0.00 | 0.00 |
| 19 | M | 15.3 | Mild | 0 | 0 | 40 | 9.4 | 57.06 | 356.94 |
| 20 | F | 4.4 | Mild | 0 | 0 | 38 | 28.9 | 79.66 | 112.13 |
| 21 | F | 5.5 | Mild | 0 | 0 | 39.1 | 23.1 | 178.85 | 0.00 |
| 22 | F | 12.5 | Mild | 0 | 0 | 40 | 20.8 | 15.57 | 0.00 |
| 23 | M | 9.6 | Mild | 0 | 0 | 39.3 | 20.9 | 56.79 | 31.26 |
| 24 | F | 10.3 | Mild | 0 | 0 | 39.9 | <7 | 25.15 | 343.80 |
| 25 | F | 5.3 | Mild | 0 | 0 | 38.7 | <7 | 190.02 | 25.78 |
| 26 | M | 4.9 | Mild | 0 | 0 | 38 | 24.9 | 53.60 | 178.67 |
| 27 | M | 3.3 | Severe | 12 | 2 | 39.0 | 46.8 | 17.8 | 329.749 |
| 28 | M | 3.6 | Severe | 3 | 3 | - | 40.0 | 8676.54 | 5292.967 |
| 29 | F | 2.0 | Severe | 4 | 0 | 38.4 | 40.8 | 1004.91 | 1778.434 |
| 30 | M | 9.8 | Severe | 8 | 5 | 39.1 | 239.0 | 808.83 | 1110.447 |
| 31 | F | 6.0 | Severe | 8 | 6 | 39.6 | 13.5 | 6544.17 | 4984.856 |
| 32 | M | 12.3 | Severe | 12 | 7 | 38.6 | 67 | 216.08 | 460.62 |
| 33 | M | 3.4 | Severe | 6 | 2 | 39.1 | 8.9 | 104.53 | 277.90 |
| 34 | M | 2.5 | Severe | 10 | 5 | 39.6 | 49.6 | 27.54 | 82.83 |
| 35 | M | 3.4 | Severe | 13 | 8 | 39.6 | 292.2 | 745.28 | 771.81 |
| 36 | M | 3.4 | Severe | 13 | 8 | 39.6 | 214.1 | 123.01 | 148.47 |
| 37 | M | 3.4 | Severe | 13 | 8 | 39.6 | 144.8 | 88.44 | 145.64 |
| 38 | M | 3.4 | Severe | 13 | 8 | 39.6 | 56.4 | 112.11 | 156.43 |
| 39 | M | 3.4 | Severe | 13 | 8 | 39.6 | 43.6 | 195.47 | 266.15 |
| 40 | M | 3.4 | Severe | 13 | 8 | 39.6 | 32.8 | 64.50 | 30.37 |
| 41 | M | 3.4 | Severe | 13 | 8 | 39.6 | 14.2 | 23.15 | 0.00 |
| 42 | M | 8.0 | Severe | 15 | 9 | 39.5 | - | 1524.92 | 1956.72 |
| 43 | M | 8.0 | Severe | 15 | 9 | 39.5 | 406.9 | 284.29 | 680.28 |
| 44 | M | 8.0 | Severe | 15 | 9 | 39.5 | 378.8 | 298.57 | 503.04 |
| 45 | M | 8.0 | Severe | 15 | 9 | 39.5 | 391 | 566.45 | 1079.55 |
| 46 | M | 8.0 | Severe | 15 | 9 | 39.5 | 356.2 | 214.03 | 648.96 |
| 47 | M | 8.0 | Severe | 15 | 9 | 39.5 | - | 77.14 | 143.13 |
| 48 | M | 8.0 | Severe | 15 | 9 | 39.5 | 81.4 | 32.59 | 0.00 |
| 49 | M | 8.0 | Severe | 15 | 9 | 39.5 | 70.4 | 42.56 | 12.20 |

*Values from number 35-41 are from the same person, but information is from different days; values from number 42-49 are from the same person, but information is from different days.


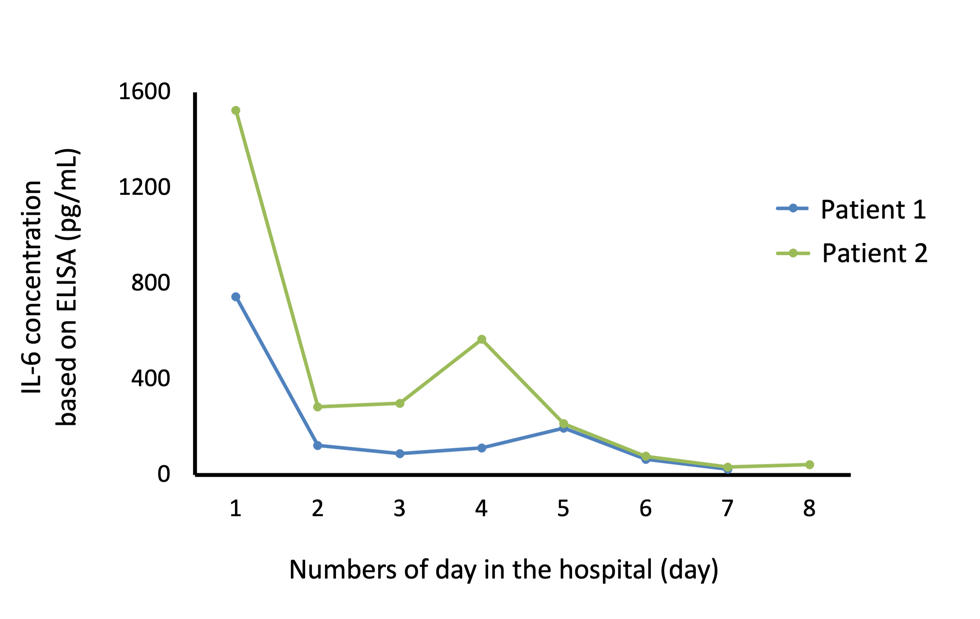


**Figure 1. Follow-up IL-6 concentrations based on ELISA compared to number of days in the hospital for two patients.**


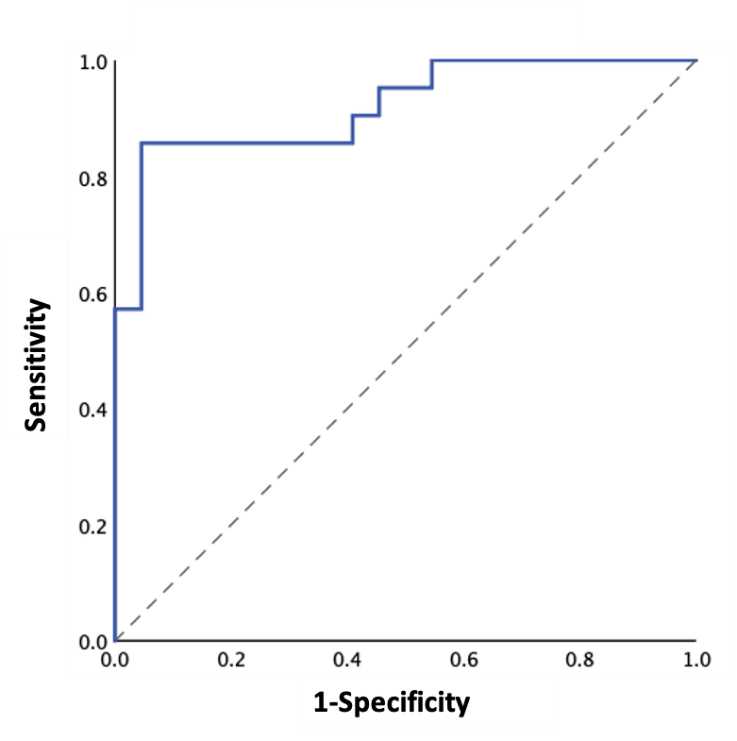


**Figure 2. ROC curve for CRP in children with influenza.**
